# From Microscopy to Nanoscopy: Defining an *Arabidopsis thaliana* Meiotic Atlas at the Nanometer Scale

**DOI:** 10.3389/fpls.2021.672914

**Published:** 2021-05-18

**Authors:** Jason Sims, Peter Schlögelhofer, Marie-Therese Kurzbauer

**Affiliations:** Department of Chromosome Biology, Max Perutz Labs, University of Vienna, Vienna BioCenter, Vienna, Austria

**Keywords:** *Arabidopsis*, meiosis, cytology, super-resolution microscopy, immunofluorescence

## Abstract

Visualization of meiotic chromosomes and the proteins involved in meiotic recombination have become essential to study meiosis in many systems including the model plant *Arabidopsis thaliana*. Recent advances in super-resolution technologies changed how microscopic images are acquired and analyzed. New technologies enable observation of cells and nuclei at a nanometer scale and hold great promise to the field since they allow observing complex meiotic molecular processes with unprecedented detail. Here, we provide an overview of classical and advanced sample preparation and microscopy techniques with an updated *Arabidopsis* meiotic atlas based on super-resolution microscopy. We review different techniques, focusing on stimulated emission depletion (STED) nanoscopy, to offer researchers guidance for selecting the optimal protocol and equipment to address their scientific question.

## Meiosis

Meiosis is a specialized cell division and the basis for genetic diversity through sexual reproduction. Understanding its molecular mechanisms and involved factors is therefore essential for human health and fertility and, importantly, for plant breeding and food security.

In contrast to somatic cells that give rise to identical daughter cells by mitotic (equational) cell division, germ cells divide meiotically to form haploid gametes, thereby ensuring constant karyotypes over generations. During the first meiotic division, after DNA replication, homologous chromosomes pair, recombine and are then separated to opposite poles of the cell. Thereafter, sister chromatids segregate during the second division. Meiosis is completed by the formation of four genetically different haploid precursor cells that develop into gametic cells.

The coordinated and tightly controlled formation of DNA double-strand breaks (DSBs) and their repair is a prerequisite for successful meiotic divisions: it ensures the pairing and segregation of homologous chromosomes as well as re-shuffling of genetic traits. Several proteins necessary for DSB formation have been identified in a variety of organisms, with the conserved topoisomerase-related protein SPO11 as the catalytically active factor within the DSB-forming complexes (reviewed in Keeney, 2001; Edlinger and Schlogelhofer, 2011; Lam and Keeney, 2014; Robert et al., 2016). SPO11-interacting proteins link sites of DSB-formation to the chromosome axis (Blat et al., 2002; Panizza et al., 2011; Acquaviva et al., 2013). The meiotic axis is formed by axial element proteins like ASY1/Hop1, ASY3/Red1, and ASY4 (Hollingsworth et al., 1990; Rockmill and Roeder, 1990; Armstrong et al., 2002; Ferdous et al., 2012; Chambon et al., 2018; West et al., 2019), together with cohesin proteins, among them SCC3 and REC8 (Klein et al., 1999; Toth et al., 1999; Cai et al., 2003; Chelysheva et al., 2005), and it is required for several processes from DSB formation to recombinational repair. Once the DSB has been formed, SPO11 is released from the DNA by the MRE11/RAD50/Xrs2-NBS1 (MRX/N) complex stimulated by COM1/Sae2 (Neale et al., 2005; Uanschou et al., 2007; Milman et al., 2009; Cannavo et al., 2018). The break ends are resected and coated with the RecA-related recombinases RAD51 and DMC1, highly conserved proteins that facilitate strand invasion of homologous sequences (Bishop, 1994; Dresser et al., 1997; Doutriaux et al., 1998; Couteau et al., 1999; Li et al., 2004; Kurzbauer et al., 2012). Subsequent DNA repair results in either crossover (CO) or non-crossover (NCO) events (reviewed in Osman et al., 2011; Hunter, 2015; Mercier et al., 2015; Sansam and Pezza, 2015), according to the selected repair template and the resolution of repair intermediates. Most organisms including *Arabidopsis thaliana*, form a large number of DSBs but only a fraction is channeled into CO recombination (Buhler et al., 2007; Hunter, 2007; Sanchez-Moran et al., 2007; Vignard et al., 2007). COs are formed in the context of the meiotic axes and the synaptonemal complex (SC) and physically link homologous chromosomes to enable correct segregation. The SC is a protein structure that builds on the axes, tightly connects homologous chromosomes and is required for inter-homolog recombination and interference (Zickler and Kleckner, 1999; Kleckner, 2006; Mercier et al., 2015; Smith and Nambiar, 2020; Capilla-Perez et al., 2021; France et al., 2021). The repair of the residual breaks yields NCO products, possibly *via* synthesis-dependent strand-annealing, intersister recombination, or non-homologous end-joining (Higgins et al., 2004; Chen et al., 2008; Mancera et al., 2008; Sims et al., 2019). Recombined homologous chromosomes segregate during the first, reductional, division, and sister chromatids during the second, mitosis-like, division, yielding four haploid cells.

## The Model Organism *Arabidopsis Thaliana*

*Arabidopsis thaliana*, or thale cress, is a small flowering plant in the mustard family and has become a widely used model organism for diverse research fields over the last decades. The weed is a simple angiosperm and has been used as a convenient model for plant biology. It is also widely used for addressing fundamental questions regarding functions common to all eukaryotes (reviewed in Meinke et al., 1998), with many factors and processes being conserved from yeast to humans and also present in plants. *Arabidopsis* plants are small, easy to cultivate under lab conditions and have a rather short life cycle of approximately 8 weeks. They are self-fertilizing and produce thousands of seeds per individual, making them especially attractive for use in genetic research. With about 135 mega base pairs, *A. thaliana* has one of the smallest plant genomes, distributed to only five chromosomes. The genome is among the best-curated ones (Berardini et al., 2015) and its near-complete sequence is available since the year 2000 (Arabidopsis Genome, 2000). We recently contributed, using latest generation sequencing approaches, considerable portions of the highly repetitive rDNA units of the nucleolus organizing region 2 (Sims et al., 2021). One of the most important advantages for the study of meiosis and related research in general is that *Arabidopsis* is thought to have very “relaxed” DNA repair checkpoints, enabling researchers to follow phenotypes of various DNA repair mutants through meiosis. In contrast to most other higher model organisms, only very few mutants (e.g., *blap75/rmi1, top3a-1*; Chelysheva et al., 2008; Hartung et al., 2008) have been identified that arrest in meiosis I and never undergo a second division. Most *Arabidopsis* mutants grow normally and complete the meiotic program regardless of accumulating DNA damage or chromosome missegregation, enabling thorough (epistatic) analyses.

These features, together with good accessibility of meiotic tissue, make *Arabidopsis* an excellent model organism to study meiosis, particularly suited for cytological analysis. There are differences between male and female meiosis (e.g., CO number; Drouaud et al., 2007; Giraut et al., 2011) and both deserve attention, but male meiocytes are more accessible because of the anatomy of *Arabidopsis* flowers. Therefore, male meiosis is typically analyzed and the focus of this review.

## Meiotic Stages (Observed Under the Widefield Microscope)

**Figure 1:**
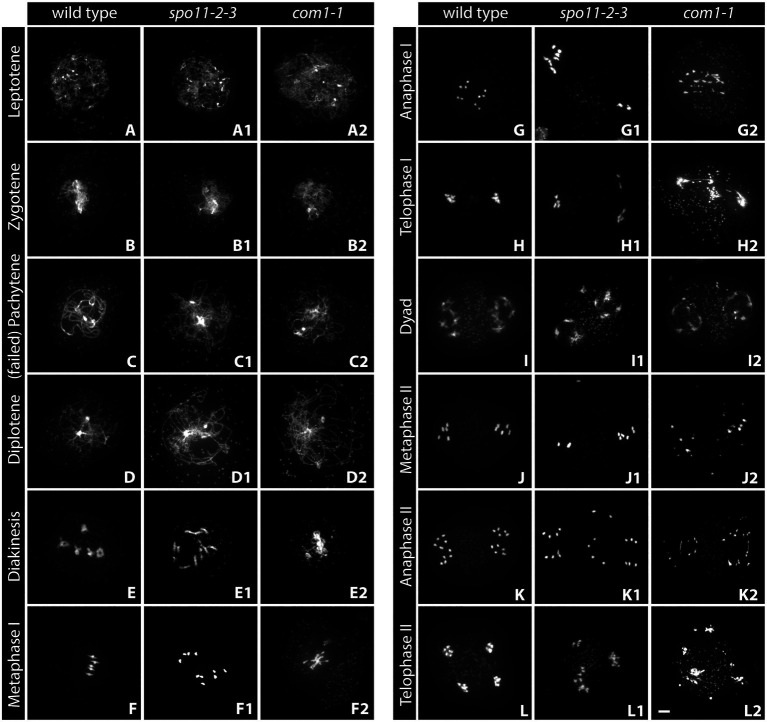
Acid-spread nuclei from pollen mother cells (PMCs) depicting the meiotic progression in wild-type, double-strand break (DSB)-deficient (*spo11-2-3*) and DNA-repair-deficient (*com1-1*) male meiocytes. The spreads were stained with DAPI and imaged with an epifluorescence microscope. See text for details. Meiotic stages are indicated. Scale Bar: 5 μm.

**Figure 2:**
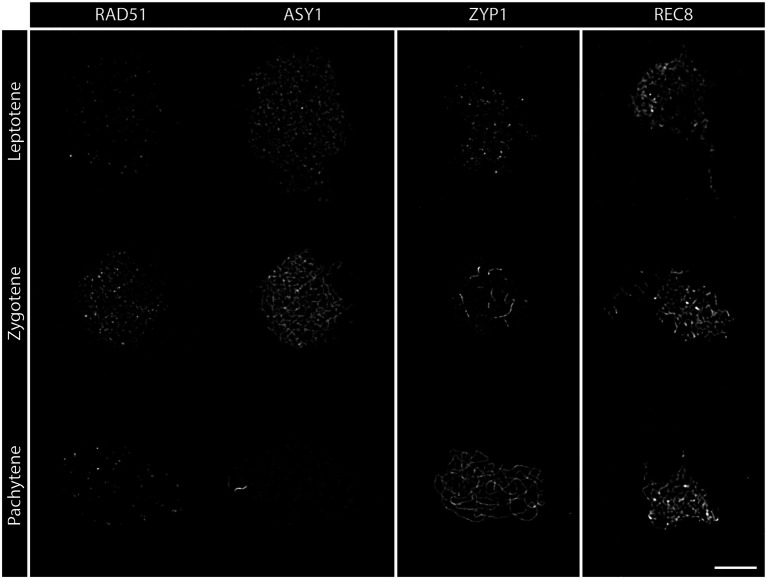
Detergent-spread nuclei from PMCs depicting the meiotic progression from leptotene to pachytene in the wild-type. The spreads were stained for the recombinase RAD51, the axial element protein ASY1, the transverse filament protein ZYP1, or the meiosis-specific cohesin subunit REC8. Images were acquired with an epifluorescence microscope. Meiotic stages are indicated. Scale Bar: 5 μm.

During **leptotene**, the first stage of meiotic prophase I, chromosomes condense after DNA replication and become visible as thin threads organized along the emerging chromosome axis. At this early stage, DSBs are formed and resected and recombinases are loaded onto ssDNA-overhangs. Leptotene nuclei can be easily identified in spreads of pollen mother cells (PMCs): thin chromatin threads are dispersed over the nucleus and the nucleolus is often visible as a darker area (Figure 1A). Leptotene is usually indistinguishable between the wild-type and DSB-deficient mutants like *spo11-2-3*, where chromosomes segregate randomly, leading to a strong reduction of fertility (Figure 1A1; Hartung et al., 2007). Likewise, DNA repair mutants like *com1-1*, which are completely sterile because DSBs are not processed and repaired (Uanschou et al., 2007), form regular leptotene meiocytes (Figure 1A2). Immunohistochemical staining reveals that the axial element proteins ASY1 and ASY3 and cohesins, such as SCC3 and REC8, are loaded during leptotene to form the axis and both recombinases, RAD51 and DMC1, appear as foci at DSB sites (Figure 2; Chelysheva et al., 2005; Ferdous et al., 2012; Kurzbauer et al., 2012; Cromer et al., 2013).

**Zygotene** is marked by the completed establishment of the meiotic axis. Chromosomes search for their homologous partner as repair templates and the SC starts to polymerize. Recombinase foci usually peak during this stage, indicating that DNA repair is in full swing (Sanchez-Moran et al., 2007; Kurzbauer et al., 2012). In acid spreads, the chromatin now appears as thicker threads partially clustered to one side of the nucleus. It is still impossible to differentiate between wild-type and DSB-deficient (*spo11-2-3*) meiocytes or nuclei lacking DNA repair factors like COM1 (Figures 1A–A2). The axial element proteins ASY1/3 appear as continuous threads over the entire length of all chromosomes in immunohistochemistry and cohesin staining is more pronounced. Staining for the SC protein ZYP1 reveals that some protein is already present on chromatin, but full polymerization will only be observed in pachytene (Figure 2). Pro-CO factors like the ZMM proteins MSH4/5 and HEI10, for example, are visible as numerous foci on chromatin (Chelysheva et al., 2012).

Complete synapsis is reached by polymerization of SC proteins from telomere to telomere and stable recombination intermediates are formed during **pachytene**. Synapsed chromosomes appear as thick, puffed up threads on acid spreads (Figure 1C) and staining for ZYP1 reveals full polymerization along chromosomes (Figure 2). ZYP1 is therefore an ideal marker when measuring total SC length within a nucleus (Drouaud et al., 2007; Lloyd et al., 2018; Kurzbauer et al., 2021). ASY1 can still be detected along pachytene chromosomes, but the staining is remarkably less bright apart from few brightly stained stretches (Figure 2) that mark the nucleolus organizer regions (NORs) containing the 45S rDNA genes (Fransz et al., 1998; Sims et al., 2019). The ASY3 signal, marking the axes now incorporated into the SC, is intense and overlaps with ZYP1 staining. The axes can also be visualized by staining for the cohesin subunits REC8 and SCC3 that appear thread-like (Figure 2). In mutants deficient for DSB formation or repair, such as *spo11-2-3* and *com1-1*, axis formation often appears to be normal, but synapsis is never complete. Pachytene stages are not found and nuclei seem to directly progress from zygotene to diplotene (Figures 1C1, 2). In these mutants, ZYP1 appears as foci or short stretches but does not fully polymerize, while axis staining is usually unaffected. Meiocytes of plants lacking axis proteins, such as ASY1 and ASY3, never fully synapse (Caryl et al., 2000; Ferdous et al., 2012) and full pachytene stages are also not observed in cohesion-deficient mutants like *scc3* and *rec8* (Bai et al., 1999; Chelysheva et al., 2005). Similarly, complete synapsis is not observed when the transverse filament ZYP1 proteins are depleted or absent (Higgins et al., 2005; Osman et al., 2006; Capilla-Perez et al., 2021; France et al., 2021). During wild-type pachytene (and up to diakinesis), ZMM proteins MSH4/5 and HEI10, as well as MLH1/3, localize to CO sites and form around 9–11 bright foci per nucleus, corresponding to the average number of chiasmata. Immunohistochemical staining for the mentioned proteins is often used to determine the number of interfering class I COs (Chelysheva et al., 2010, 2012).

**Diplotene** is the last stage of meiosis regularly amenable to analysis by detergent/surface spreading. The SC disassembles and homologous chromosomes remain linked by COs. Chromatin appears as thin, brittle, or fragmentary threads that usually occupy the whole nuclear area in acid spreads (Figures 1D–D2). Immunohistochemical images are characterized by weak axis staining.

Chromosomes condense during **diakinesis** and maximum condensation is reached during **metaphase I.** Pairs of homologous chromosomes, bivalents, align at the metaphase plate and COs are cytologically visible as so-called chiasmata (Figures 1E,F). Meiocytes are no longer compatible with detergent spreading, but acid spreading allows for further investigation of meiotic progression. Analysis of diakinesis and metaphase I chromosomes is among the first steps during the characterization of a newly found mutant, since a lot of information regarding defects can be gained. In case DSBs are not made, as in *spo11* mutants, bivalents cannot be formed and 10 univalents are observed (Grelon et al., 2001; Stacey et al., 2006; Figures 1E1,F1). The same phenotype is found in mutants deficient in interhomolog-recombination like plants lacking functional DMC1 (Couteau et al., 1999). When only a subset of interhomolog recombination events is affected, varying amounts of univalents and bivalents are observed (Higgins et al., 2004; Crismani et al., 2012; Girard et al., 2014; Kurzbauer et al., 2018). Mutant plants deficient for DNA repair like *com1-1* display aberrant chromosome behavior in diakinesis and metaphase-chromosomes are often not visible as five distinct bivalents, but rather appear as an entangled mass of chromatin (Figures 1E2,F2). Chromosome fragments may be visible as well (Bleuyard et al., 2004; Bleuyard and White, 2004). Similar defects, together with univalent formation, are often observed in plants lacking functional cohesin proteins SCC3 or REC8 (Bai et al., 1999; Chelysheva et al., 2005). Immunohistochemical staining is possible but requires special slide treatment (see section “Fluorescence *in situ* Hybridization”). Metaphase I nuclei are also analyzed to determine the number of chiasmata formed in a meiotic nucleus. Individual chromosomes are identified by fluorescence *in situ* hybridization (FISH) staining for the 5S and 45S rDNA repeats (see section “Widefield Epifluorescence Microscopy”) and the number of chiasmata per chromosome arm is deduced from bivalent shape (Sanchez Moran et al., 2001; Lopez et al., 2012).

Homologs segregate to opposite cell poles during **anaphase I** (Figure 1G) and start to decondense in **telophase I** at the end of the first meiotic division (Figure 1H). These two stages are highly informative, because chromosome fragments are visible when DNA repair is defective (as in *com1-1*, Figures 1G2,H2) and chromosome missegregation can be observed when homolog interactions are reduced or absent (as in *spo11-2-3*; Figures 1G1,H1; Grelon et al., 2001; Stacey et al., 2006). In addition, irregular repair might manifest in chromatin bridges, well visible during these (and subsequent) stages (Figures 1H2,L2; Uanschou et al., 2007; Kurzbauer et al., 2021) and cohesion-deficiencies may result in premature sister separation (Cai et al., 2003; Chelysheva et al., 2005).

The second, mitosis-like, division starts with the rather decondensed **dyad** stage (also called prophase II, Figure 1I) and chromosomes condense again during **metaphase II** (Figure 1J). Sister chromatids are then separated during **anaphase II** (Figure 1K), reach now four (or more/less in the case of missegregation as in *spo11-2-3*; Figures 1L,L1) poles in **telophase II** (Figure 1L) and finally decondense in the **tetrad** stage. Chromosome fragments and/or bridges usually persist and can be observed during all stages of meiosis II in DNA repair-deficient mutants like *com1-1* (Figures 1I2–L2).

At the end of wild-type meiosis, each originally diploid cell gives rise to four haploid gamete precursor cells (Figure 1L). Further mitotic divisions and development yield microspores (male gametes, pollen) or macrospores (female gametes, egg cells) that will reconstitute diploid organisms after nuclear fusion upon fertilization. DNA repair mutants often form polyads as a consequence of chromosome missegregation, and because chromosome fragments tend to stay highly condensed, they are visible up to the last stage of meiosis (Figure 1L2).

## Classical Sample Preparation Techniques

Male meiotic nuclei develop within anthers of small buds close to the center of *Arabidopsis* inflorescences. In order to subject them to cytological analysis to follow chromosomes through meiosis, several layers of tissue have to be removed mechanically and the syncytium-surrounding callose eliminated by digestion. Finally, remaining membranes and cytoplasm are cleared away by spreading and chromatin is spread and fixed to the slide. Two different sample preparation techniques – and several variations thereof – are used for most cytological studies, depending on the specific research question (see below).

### Acid Spreads

Production of acetic acid spreads of PMCs followed by chromatin staining is a standard technique to analyze meiotic progression and assess chromosome structure and segregation in male *Arabidopsis* meiocytes since the 1990s (Ross et al., 1996). The technique has been refined over the years but the basic procedure (destaining and fixation in acetic acid/ethanol, enzymatic digestion of the cell wall and spreading with acetic acid/ethanol) is still the same (Fransz et al., 1998; Armstrong et al., 2001). The procedure is rather simple, required reagents and equipment are widely available. It is usually the starting point for studying mutant phenotypes related to meiosis. Preparation of acid spreads allows for a relatively quick assessment of which stage of meiosis is defective in a mutant of interest (see section “Classical Sample Preparation Techniques”). Several years ago, this spreading technique was developed further to allow for the staining of proteins constituting the axis and/or the SC as well as closely associated factors. Like the original, this refined method preserves chromosome structure by strong fixation and removes cytoplasm by acetic acid treatment. It includes additional microwave treatment to increase the accessibility of epitopes to antibodies, thereby allowing for immunostaining of nuclei in all stages of meiosis (Chelysheva et al., 2010, 2012, 2013).

### Detergent Spreads

Detergent spreading followed by immunohistochemical staining with antibodies directed against meiotic proteins and fluorescently labeled secondary antibodies for visualization is used for a more detailed analysis of events during early-to-mid meiotic prophase. It enables investigation of the temporal expression and localization of proteins from leptotene to diplotene when antibodies are available. The technique for male plant meiocytes was originally developed for electron microscopic analyses in the Jones lab (Albini et al., 1984) and has been optimized over the years (for example, Armstrong et al., 2002; Chelysheva et al., 2005; Kurzbauer et al., 2012; Armstrong and Osman, 2013; Martinez-Garcia et al., 2018; Sims et al., 2020b). The original basic procedure involves preparation of anthers containing meiotic cells, enzymatic digestion (for example, with cytohelicase, adigestive enzyme from *Helix pomatia* containing several enzymatic activities required to digest the callose surrounding meiotic syncytia), chromatin spreading with a detergent (often Lipsol) and formaldehyde fixation on glass slides. The protocol yields differing amounts of meiotic nuclei on slides, since the exact stage of meiocytes in the anthers is hard to predict and some material is lost during preparation. Additionally, the non-meiotic tissue of anthers present on the slides leads to high amounts of background staining. To overcome this, we previously took advantage of a technique developed by Chen et al. (2010). Meiotic nuclei develop within four elongated syncytia per anther (six anthers per flower) that can be separated from the surrounding tissue. Mechanical extraction and subsequent collection of those “columns” with a glass capillary allows for enrichment of meiotic nuclei in very small volumes and greatly improved the quality of microscopic preparations (Kurzbauer et al., 2012; Sims et al., 2020b). The preparation furthermore removes all non-meiotic cells, improving digestion and spreading efficiency, reducing background staining and increasing the amount of analyzable nuclei per microscopic slide. This reduces the time spent at the microscope considerably and enables more thorough analyses by super-resolution microscopy (see below).

## Advanced Sample Preparation Techniques

Specific research questions often demand a precise localization of molecular events to defined chromosomal locations, with respect to the entire genome or to its spatial position within the nucleus. Such analyses are enabled by the techniques presented below that were developed over the last decades with the contribution of many researchers.

### Fluorescence *in situ* Hybridization

Fluorescence *in situ* hybridization or FISH is a cytological technique developed more than 35 years ago (Bauman et al., 1980) and widely used to specifically mark nucleic acid sequences in chromosome preparations. It has a wide range of applications from individualizing each chromosome to monitoring specific chromosomal regions. The principle of FISH has not changed since its first application but the protocols and techniques used to prepare the samples and the hybridization probes have improved over the years. In brief, the fixed spread chromosomes (see section “Fluorescence *in situ* Hybridization”) are heat-denatured in order to allow fluorescently labeled nucleic acid probes to hybridize to their DNA target. The nucleic acid probes can be “self-made” by synthesizing DNA from template sequences and incorporating fluorescent base analogs, or by using custom-made, commercially available labeled locked nucleic acid (LNA) sequences (Paulasova and Pellestor, 2004). LNA probes have the great advantage to have a strong affinity to DNA (or RNA) and can bind to their target at lower temperatures than regular DNA-only probes. Recent techniques combine bioinformatic platforms and PCR-based oligonucleotide labeling to allow imaging of regions from tens of kilobases to megabases (Beliveau et al., 2012). In addition, pre-labeled oligomer probes (PLOPs) were used to mark repetitive regions of different plant species and have the potential of reducing the hybridization time from hours to minutes (Waminal et al., 2018). The FISH technique has been widely used within the meiotic plant community and is an essential tool to determine the chiasma frequency on individual chromosomes. To this end, bivalents are unequivocally identified by FISH labeling of the 5S and 45S rDNA regions and the number of chiasmata per chromosome arm is deduced from bivalent shape (Sanchez Moran et al., 2001; Lopez et al., 2012; Armstrong, 2013; Kurzbauer et al., 2018, 2021). Finally, single-molecule RNA-FISH has become increasingly popular to analyze transcription in plant tissues. It involves the use of fluorescently labeled DNA probes that bind multiple times within a single mRNA transcript (Femino et al., 1998; Duncan et al., 2016). This technique is routinely used on root meristems but, to our knowledge, has not yet been optimized for meiotic cells (Duncan and Rosa, 2018).

### Targeted Analysis of Chromatin Events

The Targeted Analysis of Chromatin Events (TACE) is an advanced cytological method that combines an improved immunocytology protocol (detergent spreads; see section “Targeted Analysis of Chromatin Events”) with the hybridization of FISH probes targeting large chromosomal regions (Sims et al., 2020b). Thereby, the localization and abundance of meiotic proteins can be determined at specific chromosomal loci of interest. Regular FISH on acid-spread chromosome preparations is used to visualize any desired chromosome locus but the harsh preparation followed by heat denaturation of the DNA/RNA can cause mis-folding or loss of proteins and failure of detection. It is therefore important to fine-tune the denaturation and hybridization steps to detect both the proteins of interest and the desired chromosomal loci. For this specific application, LNA probes are favorable due to their low hybridization temperatures. TACE has been used recently to determine the abundance of RAD51 DNA repair foci in defined regions with and without an ectopic rDNA insertion (Sims et al., 2019). It can be easily adapted to specific research questions by combining different sets of antibodies and nucleic acid probes.

### Whole Mount Immuno FISH

Whole mount immunolocalization is a popular sample preparation technique when the proteins of interest are to be analyzed in 3D preserved tissues and nuclei and is especially recommended for visualizing female *Arabidopsis* meiocytes (Escobar-Guzman et al., 2015). Tissue clearing may be necessary to improve protein detection and has been implemented in studies of both female and male meiosis (Hedhly et al., 2018; Tofanelli et al., 2019). FISH is usually performed on spread samples (see previous sections). Specific questions, however, may require preserving the spatial organization of the cell and the relative position of genomic loci or protein complexes within the nucleus. Whole mount FISH protocols were developed that maintain the structural integrity of nuclei, cells and even tissues in 3D and let researchers address the original spatial relations. Several protocols are currently available and have been optimized for different tissues: a necessity, since a caveat for whole mount preparations is the difference in cell wall composition and thickness affecting the penetrance of antibodies and probes (Bauwens et al., 1994; Bass et al., 1997; Costa and Shaw, 2006; Berr and Schubert, 2007; Bey et al., 2018). The special whole mount immuno FISH (Who-M-I-FISH) technique has been optimized for the simultaneous detection of meiotic proteins and genomic loci, while maintaining the 3D structure of meiotic nuclei within intact anthers (Sims et al., 2020a). It has been used recently to determine the 3D localization of the rDNA regions within the nucleus in relation to the localization of the HORMA domain protein ASY1 (Sims et al., 2019). The long duration of the protocol and the often incomplete penetrance of the primary antibodies through the plant cell wall make the Who-M-I-FISH a challenging technique that should only be considered when addressing specific questions.

## Microscopy Techniques

The wide range of microscopy technologies that were developed in the last decade, together with long approved techniques, offers a large choice of microscopes that can fit any lab requirement. In the meiotic field, cytological analysis is a necessary and widely used tool for all model organisms.

### Widefield Epifluorescence Microscopy

Widefield epifluorescence microscopy uses a basic illumination principle that permanently illuminates the whole sample and detects emitted light with a digital camera. It can be considered as the “workhorse” technology that enables a first screen of the samples and a general overall assessment of sample quality and staining efficiency. Widefield microscopy is regularly used to analyze and image acid spreads, FISH preparations and detergent spreads with all of the previously described improvements. This technology is still the prime candidate for quantitative analysis due to its user-friendly set up and the affordable price. The X-Y resolution of this technology is limited by diffraction to ~200 nm, and the axial resolution to about 500–700 nm (Verdaasdonk et al., 2014; Kubalová et al., 2021). The possibility to deconvolve and further process the acquired images can improve resolution and image quality. For these reasons, widefield epifluorescence microscopy is still the system of choice for most applications.

In order to achieve higher resolutions some microscopy technologies make use of sophisticated optics and algorithms to surpass the physical ~200 nm diffraction limit (Biggs, 2010; Kubalová et al., 2021).

### Confocal Laser Scanning-Airyscan Microscopy

Confocal Laser Scanning-Airyscan microscopy or LSM-Airyscan uses a new detector concept, developed by the company Zeiss, implemented on confocal laser-scanning microscopes. Canonical confocal microscopes scan specimens point-by-point, using point illumination and a pinhole at the detector level to eliminate out-of-focus light. In addition, the LSM-Airyscan has a 32-channel detector that collects 32 pinhole images with positional information at every scan point. This enables very light-effective imaging with improved resolution. According to the manufacturer, and based on imaging of fluorescent beads, the Airyscan detector system can reach a super-resolution of 120 nm in the x-y and 350 nm in the z plane even when scanning thick samples (Huff, 2015; Huff et al., 2017; Kubalová et al., 2021). This makes the LSM-Airyscan the microscopy technique of choice when imaging thick samples, such as whole mount preparations, although more complicated to operate. The LSM-Airyscan system relies on algorithms to reconstruct the image and achieve super-resolution, which can cause artifacts (Korobchevskaya et al., 2017). Image acquisition by confocal laser scanning microscopy is rather fast, particularly suited for live imaging of thick samples. In fact, it is one of the best-suited technologies for live imaging of meiotic cells in intact tissues (Prusicki et al., 2019). Alternatively, **light sheet fluorescence microscopy** may be employed to follow meiotic progression live (Valuchova et al., 2020). Here, the sample is excited with a thin sheet of laser light and optical sections are captured. Acquisition speed and low phototoxicity allow for extended imaging periods with a large field of view, but, being diffraction-limited, the resolution cannot compete with LSM-Airyscan systems.

### Structured Illumination Microscopy

Structured Illumination Microscopy (SIM) illuminates the sample using patterned light at different focal planes. Multiple images of the different light patterns are combined by a computer algorithm to reconstruct a super-resolved image. SIM microscopy can reach a resolution of 100 nm in the x-y and 350 nm in the z plane and it is one the most widely used super-resolution techniques (Kubalová et al., 2021). SIM is generally easy to use and suitable for a wide variety of samples, although it is not optimal for thick samples; it is based on widefield microscopy and fails in the presence of excessive out-of-focus light (Cox, 2015). Furthermore, because it uses algorithms to reconstruct the image, there is a chance of generating artifacts in the final image. Hammer-stroke or honeycomb-like artifacts are most common and can be mistaken for biological structures (Schaefer et al., 2004; Komis et al., 2015; Lambert and Waters, 2017; Sivaguru et al., 2018; Kubalová et al., 2021).

In general, SIM is used for 3D reconstructions of completely or partially spread samples allowing a more detailed analysis of the localization of proteins. Many labs routinely use SIM and contributed to the optimization of the sample preparation for an optimal performance (for example, Schermelleh et al., 2008; Lambing et al., 2015; Hesse et al., 2019; Mittmann et al., 2019; Ku et al., 2020; Morgan and Wegel, 2020). SIM has been used to study many different aspects of meiotic cells from the structure of the axis/synaptonemal complex to the architecture of centromeres (Lloyd et al., 2018; Morgan et al., 2020; Schubert et al., 2020).

### Stimulated Emission Depletion Microscopy

Stimulated emission depletion or STED microscopy is a technology based on laser scanning confocal microscopy but uses a depletion laser, bearing a donut shape, in conjunction with the excitation laser beam. The depletion laser de-excites fluorophores, which are not at the center of the donut, thereby preventing a spontaneous emission and creating an extremely small photon-emitting spot. The fluorescence at the periphery of the excited spot is silenced, thereby improving the resolution far beyond the diffraction limit (Hell and Wichmann, 1994; Klar and Hell, 1999). STED nanoscopy has similar requirements as classical confocal microscopy but with some differences in sample preparation. Specific fluorophores are required to achieve the best resolution. These fluorophores need to withstand the power of the depletion laser and resist its bleaching capacity. Furthermore, the mounting medium used on the samples can severely alter imaging quality. Hence, extra caution must be taken when preparing the sample for STED imaging.

Fluorophores emitting in the far-red wavelength (700–800 nm) can reach up to 30 nm of resolution, in contrast to red wavelength emitters (620–750 nm), which can reach a maximum of 50–60 nm (in the X-Y plane, maximum axial resolution is around 90 nm; Kubalová et al., 2021). This discrepancy in the resolution between the two wavelengths needs to be taken into account when performing protein co-localization studies. A great advantage of STED nanoscopy, if compared to other super-resolution techniques, is that the final acquired imaged is not the result of algorithm-based image reconstruction, but rather of a purely physical improvement in resolution. This minimizes artifacts and maintains image fidelity (Lambert and Waters, 2017). STED nanoscopy is a recent technology and has not yet been widely adopted by the meiosis community but has been used to study the length of DNA loops, the width and structure of the synaptonemal complex and precise abundance of meiotic proteins at specific sites (Sims et al., 2019; Capilla-Perez et al., 2021; Kubalová et al., 2021; Kurzbauer et al., 2021).

The basic STED set-up is not well suited for live imaging owing to the requirement of the high-intensity depletion laser. This can result in a reduction of cell fitness, but new techniques are being developed that will improve super-resolution live cell imaging in the future (Sharma et al., 2020).

One of the great advantages of the STED technology, which allowed the system to jump into the microscopy market, is the capacity to upgrade existing microscopes to a STED system with an affordable basic version (Abberior STEDycon). This unfortunately comes with some limitations such as a restriction in the number of channels that can be imaged in super-resolution and only in 2D. Bigger and more costly setups overcome these limitations providing super-resolution in more channels and in 3D.

Certainly, there are very powerful other super-resolution technologies available, such as **photo-activated localization microscopy** (PALM) or **stochastic optical reconstruction microscopy** (STORM), which can reach a resolution of 10 nm (Komis et al., 2018). Both are widefield techniques and rely on the detection of individual fluorophore molecules to overcome the diffraction limit. In short, fluorophores are sparsely activated and forced into “blinking” by spontaneous photobleaching (PALM) or reversible On/Off switching (STORM). A large number of images are generated, where each contains signals emitted by a different set of fluorescent molecules, enabling their precise localization. The complexity of the sample preparation and the inherent post-acquisition processing, required to reconstruct the final image, can be prone to artifact generation (Whelan and Bell, 2015; Lambert and Waters, 2017). For these specific reasons, the STED technology is often preferable when analyzing meiotic spreads in super-resolution and with maximum image fidelity.

Overall, the possibility for every lab to upgrade any existing epifluorescence microscope with commercially available STED technology brings the possibility to use super-resolution imaging on a daily basis.

## From Microscopy to Nanoscopy: New Views on Meiosis

### Super-Resolution Meiotic Atlas

**Figure 3:**
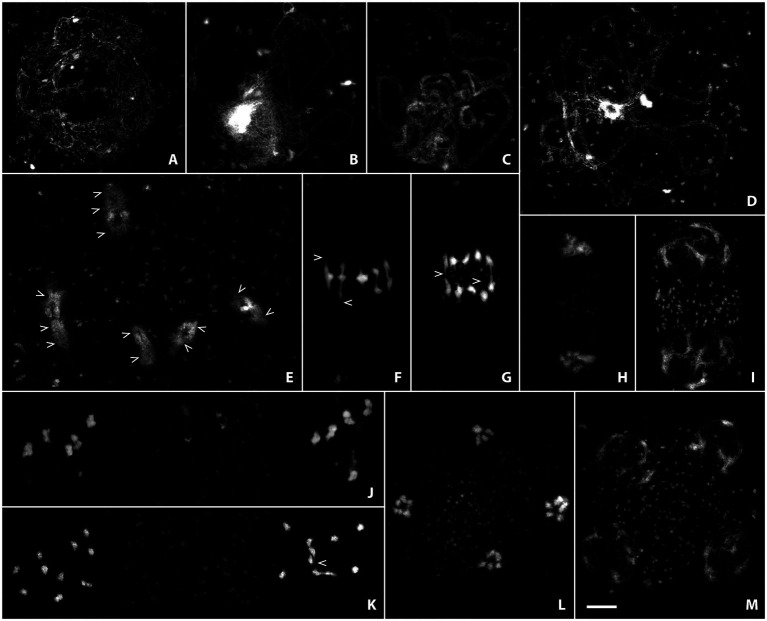
Acid-spread nuclei from pollen mother cells depicting the meiotic progression in wild-type male meiocytes in super-resolution. Chromatin was stained with SiR DNA and imaged with a stimulated emission depletion (STED) nanoscope. The meiotic stages are: **(A)** Leptotene; **(B)** Zygotene; **(C)** Pachytene; **(D)** Diplotene; **(E)** Diakinesis; **(F)** Metaphase I; **(G)** Anaphase I; **(H)** Telophase I; **(I)** Dyad; **(J)** Metaphase II; **(K)** Anaphase II; **(L)** Telophase II; **(M)** Tetrad. Scale Bar: 5 μm.

The first complete “atlas” of meiosis in *A. thaliana* was published in 1996 and encompassed images of acid-spread pollen mother cells in all stages from pre-leptotene to telophase II. The idea of the authors was to provide a reference of normal chromosome behavior and appearance in wild-type meiosis against which mutant phenotypes could be compared, using “simple light microscopic techniques” (Ross et al., 1996). About 25 years later, we present an updated meiotic atlas, combining classical slide preparation by acid spreading (see section “Fluorescence *in situ* Hybridization”) with the rather new chromatin dye SiR-Hoechst (SiR-DNA; Lukinavicius et al., 2015) and state-of-the-art STED nanoscopy (Figure 3; Supplementary Material). Acid spreading is extremely efficient and, together with the use of an optimized chromatin dye and a STED nanoscope, enables the observation of meiotic chromosomes at a nanometer scale, revealing fascinating details and opening possibilities for new analyses.

The size of chromatin loops, for example, can be measured in euchromatic and heterochromatic regions from leptotene to diakinesis (see Figures 3A–E; Kurzbauer et al., 2021) without the requirement for additional FISH labeling or electron microscopy, that had been used previously to measure the length of chromatin loops of meiotic chromosomes (Anderson et al., 1988; Moens and Pearlman, 1988; Heng et al., 1996; Novak et al., 2008). Synaptonemal complex length and loop size are inversely correlated through loop density (Kleckner, 2006), a parameter that can be determined fairly easily using the above described techniques. Pachytene nuclei are furthermore often spread well enough to distinguish chromatin from the two synapsed homologous chromosomes (Figure 3C), opening the possibility to screen for unpaired or mispaired regions that might be caused by translocations or other chromosomal aberrations. During diakinesis, chiasmata (the cytologically visible form of crossovers) can be directly observed and their number approximated without the use of further markers (Figure 3E; arrowheads). Delicate chromatin protrusions are visible during diakinesis and metaphase I (Figures 3E,F; examples marked by arrowheads), and connections between segregating chromosomes by crossover recombination can be seen well into anaphase I (Figure 3G; examples marked by arrowheads). Early on in meiosis II, before sister chromatids have really separated, they already become visible as discrete chromatin entities (Figure 3J). Fine connections may be observed in anaphase II (Figure 3K; arrowhead) before sisters finally segregate to separate poles, and chromosomes progressively decondense during telophase II and the tetrad stage (Figures 3L,M).

Imaging at such high resolution on classically prepared slides has only recently become available and will be very useful for future studies. Meiotic progression of mutant meiocytes can now be studied in greater detail, since very subtle defects are more obvious when imaged in super-resolution. Even small chromosomal fragments may be identified, premature sister separation can be observed, thin chromosome bridges become visible, and changes in loop size may be assessed.

### The Meiotic Axis and the Synaptonemal Complex in Super-Resolution

**Figure 4:**
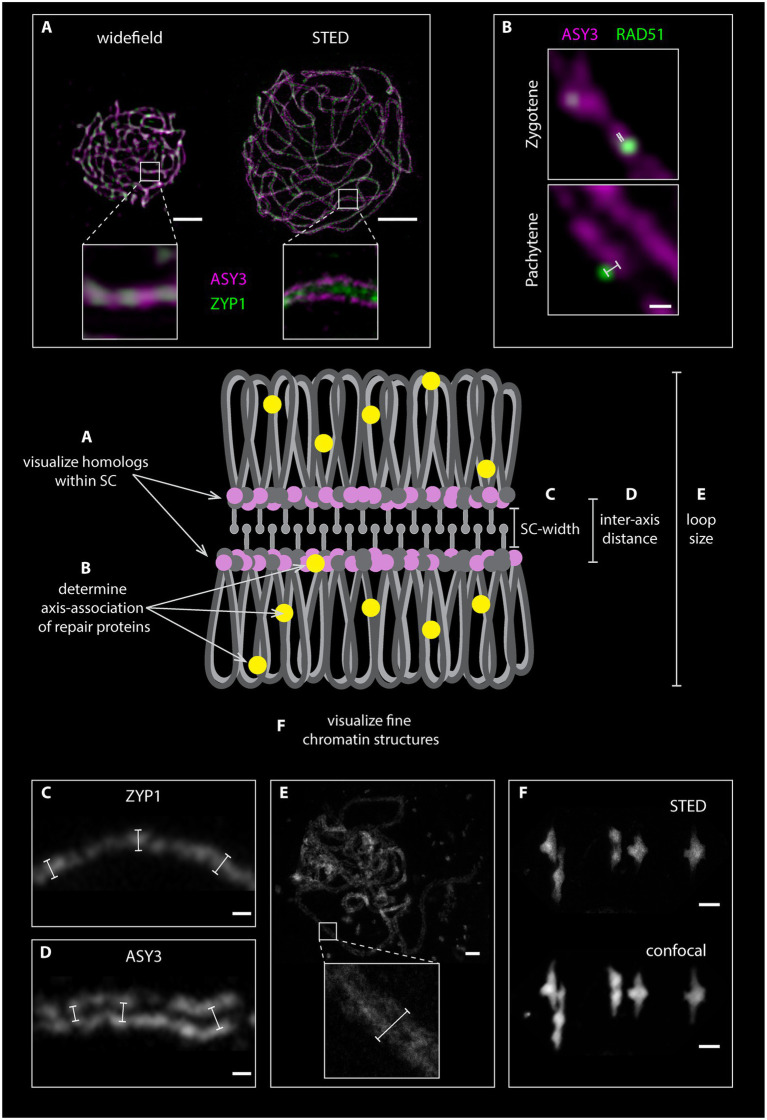
Graphical representation of homologous chromosomes with a fully formed synaptonemal complex (SC). The loops of the homologous chromosomes are depicted in gray, the axial elements in pink and dark gray, the SC transverse filaments in light gray and repair proteins in yellow. Chromosomal features, which can be assessed by STED nanoscopy, are highlighted. **(A)**. Comparison between widefield and STED images of detergent-spread male meiotic nuclei, which were stained for the axis (ASY3) in magenta and the transverse filament (ZYP1) in green. **(B)**. Magnification of meiotic chromosomes in zygotene and pachytene stage stained for ASY3 (magenta) and the recombinase RAD51 (green). White bars indicate the distance measurements of each focus from the center for the axis. **(C)** and **(D)** Examples of measurements of SC width and inter-axis distance in nuclei stained for ZYP1 **(C)** and ASY3 **(D)**. **(E)** Measurement of DNA loop length on an acid spread pachytene nucleus. **(F)**. Comparison between STED and confocal microscopy of an acid-spread nucleus at metaphase I stained with SYBR green (confocal) and SiR DNA (STED). Scale Bars: **(A,E,F)** 2 μm; **(B,C,D)** 100 nm.

**Figure 5:**
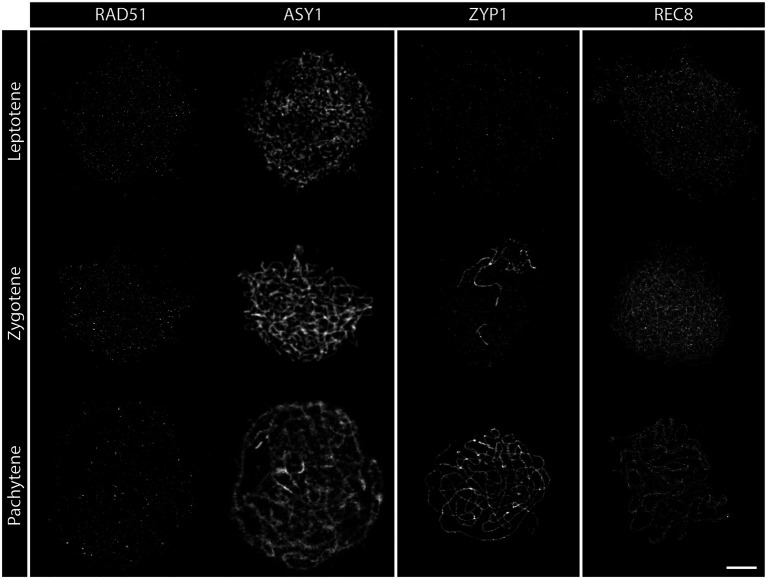
Detergent-spread nuclei from pollen mother cells depicting the meiotic progression in the wild type. The spread nuclei were stained for the recombinase RAD51, the axial element protein ASY1, the transverse filament protein ZYP1, or the cohesin subunit REC8. Images were acquired with a STED nanoscope. Stages of meiotic prophase are indicated. Scale Bar: 2 μm.

**Figure 6:**
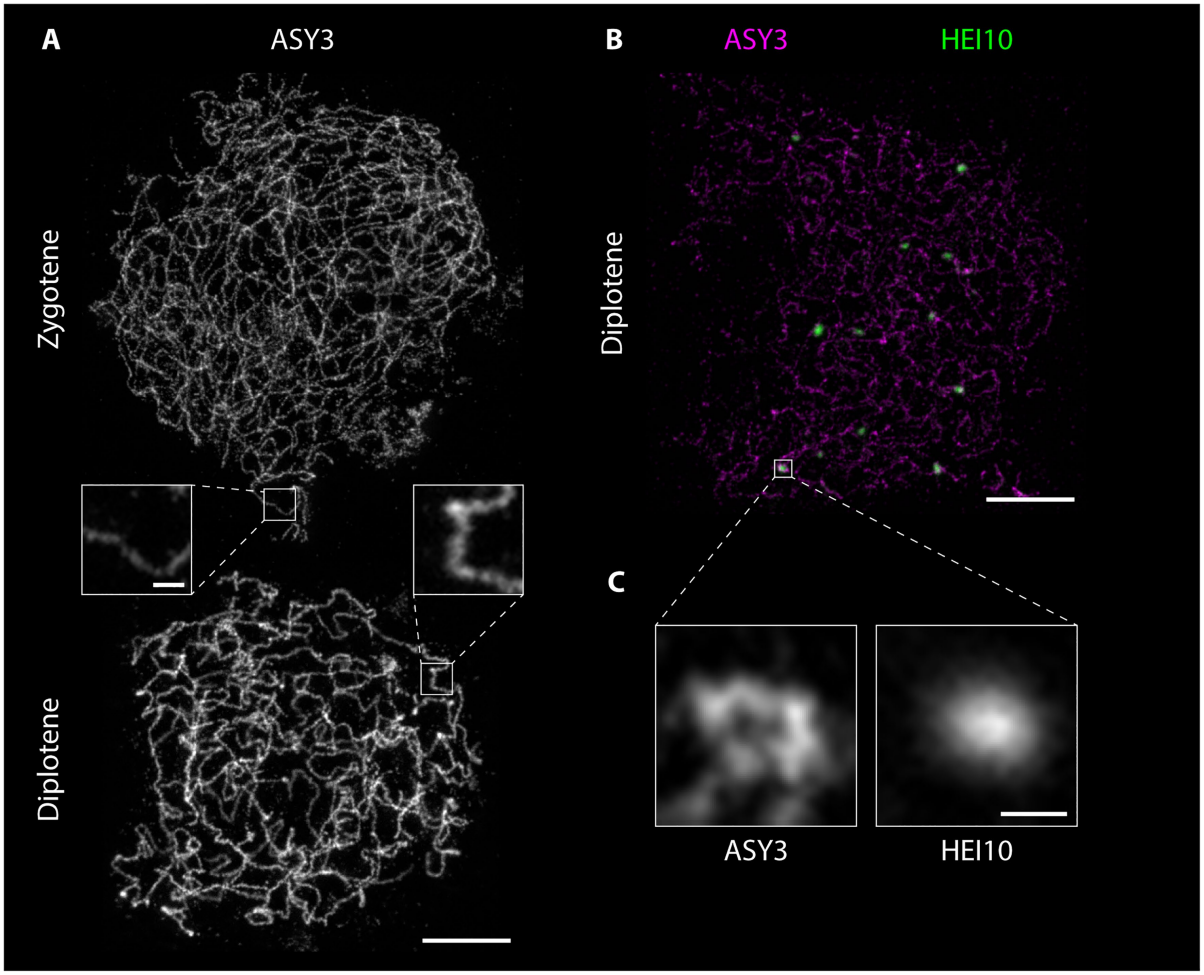
Detergent-spread male meiotic nuclei were stained for the axial element protein ASY3 **(A)** or ASY3 and the ubiquitin ligase HEI10 **(B)** and imaged with a STED nanoscope. Scale Bar: 2 μm. Panel **(C)** shows a magnification of the highlighted region in panel **(B)**. Scale Bar: 200 nm.

Preparation of detergent-spread meiotic nuclei, together with immunofluorescence staining and imaging by STED nanoscopy, offers new possibilities to study basic chromosomal structures, especially important for model organisms with rather small chromosomes like *Arabidopsis*. The structure of the SC (mainly studied using electron microscopy) appears to be highly conserved with a width of around 100 nm in many organisms with vastly different genome and chromosome sizes (e.g., Zickler and Kleckner, 1999). With 100 nm or more being the resolution limit for other super-resolution techniques (see section“From Microscopy to Nanoscopy: New Views on Meiosis”), imaging substructures of the SC is an ideal case for STED nanoscopy: in pachytene, ASY3 staining is intense and appears as parallel threads around the ZYP1 signal, while the localization of both proteins appears almost identical in epifluorescence images (Figure 4A; Supplementary Material). It is therefore possible to not only visualize both axes embedded in the SC of synapsed homologs, but also to measure the distances between them, providing a novel parameter for defining meiotic chromosomes (Figures 4C–E; Supplementary Material). Such measurements only recently revealed that the *Arabidopsis* SC is roughly 125 nm wide, that axis midpoints are about 140 nm apart, and that those parameters depend on regulatory proteins (Kurzbauer et al., 2021). The homologous axes can also be distinguished when staining for cohesins like REC8 and SCC3 that appear as abundant, close-packed foci alongside transverse filaments, in contrast to thick lines observed with widefield microscopy (compare Figures 2, 5). STED imaging of *Arabidopsis* nuclei also reveals that extensive chromosome axis remodeling, in preparation for higher condensation during subsequent stages, results in axis structures highly similar to those observed in “tinsel-like” stages in the large genome cereals barley and wheat (Colas et al., 2017). Instead of the curved threads visible during zygotene or pachytene, the ASY3-stained axes appear as short, straight, and thicker stretches with kinks in between (Figure 6A; Supplementary Material). Nanoscopic analysis of *Arabidopsis* detergent spreads furthermore shows that the axis opens up around HEI10-labeled recombination sites, forming pocket-like substructures, in diplotene (Figures 6B,C; Supplementary Material), similar to previous observations in *Caenorhabditis elegans* (Woglar and Villeneuve, 2018). Since antibodies directed against numerous components of meiotic chromosomes are available, future nanoscopic studies will shed light on previously unknown (or rather unseen) substructures and protein (co-)localization and refine our understanding of basic meiotic processes.

### Meiotic Repair Proteins and Co-localization of Complex Partners

The abundance and localization of meiotic repair proteins have been predominantly addressed using epifluorescence microscopy. More recently, SIM, and in some cases STORM, have been used to analyze the dynamics of proteins throughout meiotic prophase (Brown et al., 2015; Woglar and Villeneuve, 2018; Hinch et al., 2020; Morgan and Wegel, 2020; Slotman et al., 2020).

The nanoscale resolution of 30 nm in STED nanoscopy allows analyzing the position and dynamics of proteins with nanometer precision in a qualitative and quantitative manner. Furthermore, the absence of image reconstruction alleviates the concern of generating artifacts. The most striking difference between the canonical epifluorescence microscopy and STED nanoscopy in terms of protein localization by immunofluorescence is in the number of detected proteins. Each single focus found at an epifluorescence microscope is composed of several smaller foci at the STED (compare RAD51 staining in Figures 2, 5). While RAD51 foci, for example, were found to be mainly circular and peak in zygotene at around 200 foci per nucleus in widefield images (Kumar et al., 2019; Sims et al., 2019; Kurzbauer et al., 2021), more than 1,000 foci are found in images acquired by STED nanoscopy (Figure 5). In addition, foci seem to assume different shapes over time, with few larger clusters forming in pachytene and likely representing different repair intermediates. In this sense, most of the measurements made in terms of numbers and shapes of proteins at the epifluorescence microscope need to be re-evaluated at the STED.

**Figure 7:**
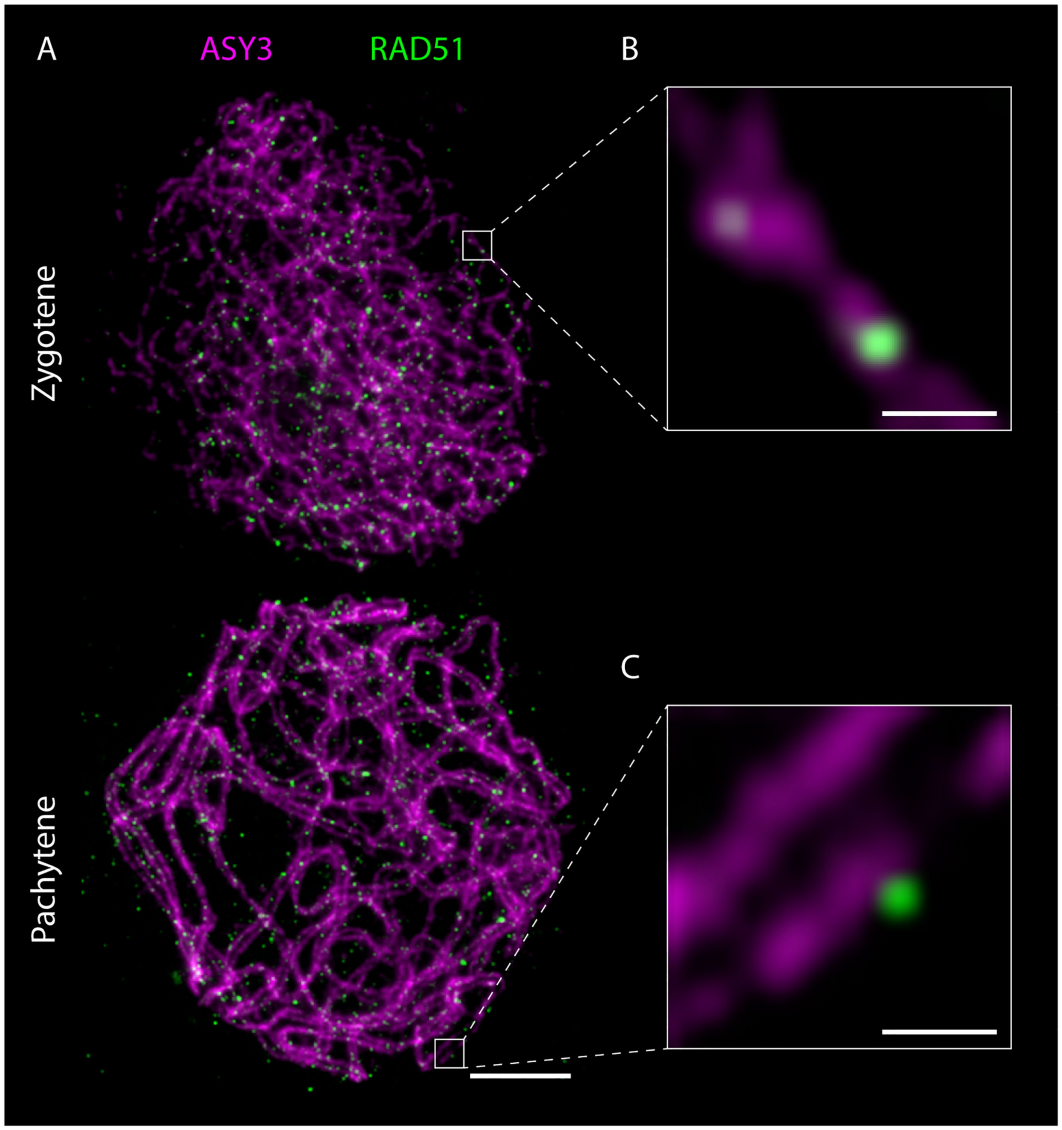
Detergent-spread male meiotic nuclei were stained for the axial element protein ASY3 and the recombinase RAD51 and imaged with a STED nanoscope. Scale Bar: 2 μm. Panels **(B)** and **(C)** show magnifications of the highlighted regions in panel **(A)**. Scale Bars: 200 nm.

The nanoscale resolution provided by STED imaging furthermore allows precise localization of proteins within the meiotic nucleus and in relation to other proteins, the chromatin loops or other substructures. These new parameters should be taken into account in future studies and will help to characterize meiotic players and their function. New insights can be gained by observing the dynamics of specific proteins and their relation to the chromosome axis, where DSBs are thought to form. The axis association of RAD51, for example, changes throughout meiotic prophase (Figures 5, 7; Supplementary Material), with the recombinase being initially located on (or in close proximity of) the ASY3-labeled axis in leptotene/zygotene stages and then further apart in pachytene (Figures 7B,C; Supplementary Material), when homolog invasion is completed. Similar dynamics can be expected for further repair proteins.

Another interesting aspect amenable to analysis by STED nanoscopy is the possibility to address the co-localization of complex partners. At a spatial resolution of 30 nm, proteins that appear to cover the entire axis in widefield images appear as individual and defined foci (compare REC8 staining in Figures 2, 5) in STED-acquired images. This holds promise to reveal more complex relationships between proteins and requires a redefinition of co-localization for future studies. When measuring co-localization between proteins one important caveat needs to be taken into consideration: the measurements could yield deviating results depending on the primary antibody used for the analysis. This is because each primary antibody might recognize different epitopes of the same protein, and this can be resolved at a resolution of 30 nm, as shown in a recent publication (Capilla-Perez et al., 2021). Furthermore, an additional variation is added to the measurements if the combination of primary and secondary antibodies, which is roughly 30 nm long, is taken into account.

## Concluding Remarks

The advent of super-resolution microscopy has dramatically changed the way, we analyze and acquire our images. This has generated a new line-up of parameters to consider which in turn generate novel and unexpected results. The use of STED nanoscopy for acquiring qualitative and quantitative data will provide a portfolio of parameters to be analyzed: while the described technique certainly opens new possibilities for mutant analyses, it will also serve to better understand wild-type meiosis. In fact, with new technologies come new insights, which will shed new light on old problems. Without a doubt, the advancements in technology and the improvement of the different microscopy techniques will push the boundaries of our current knowledge and promote exciting new revelations.

## Author Contributions

JS, PS, and M-TK: conceptualization and writing. JS and M-TK: visualization. PS: funding acquisition. All authors contributed to the article and approved the submitted version.

### Conflict of Interest

The authors declare that the research was conducted in the absence of any commercial or financial relationships that could be construed as a potential conflict of interest.

## Supplementary Material

Supplementary Material Poster version of Figures 3, 4, 6, 7.
