# Supplementary material for: From Microscopy to Nanoscopy: Defining an Arabidopsis thaliana Meiotic Atlas at the Nanometer Scale
Source: Front Plant Sci. 2021 May 18;12:672914. doi: 10.3389/fpls.2021.672914 (PMC8167036; doi:10.3389/fpls.2021.672914)

From microscopy to nanoscopy: defining an *Arabidopsis thaliana* meiotic atlas at the nanometer scale

Jason Sims, Peter Schlögelhofer and Marie-Therese Kurzbauer

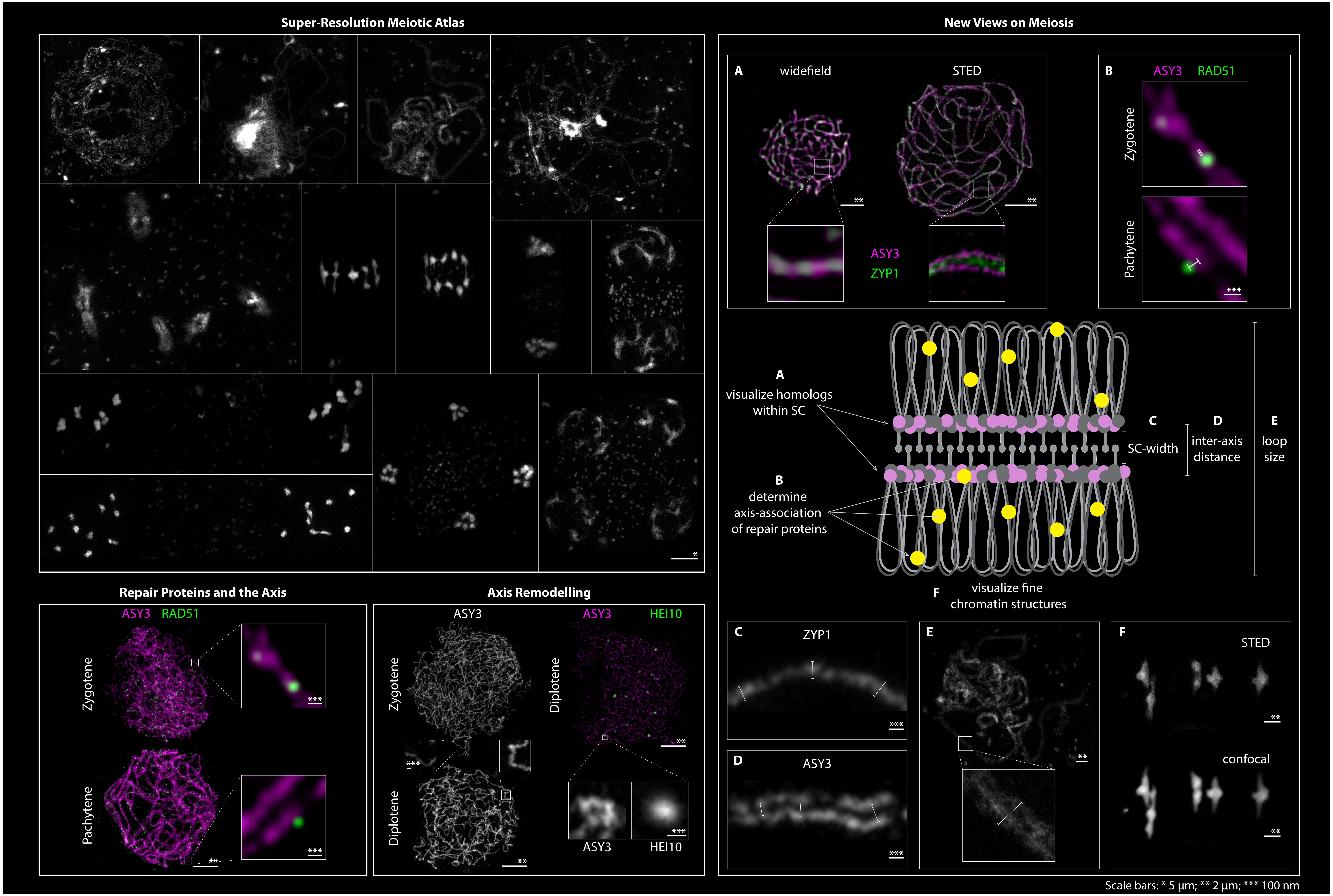

Supplement: Supplementary Material — Poster version of Figures 3, 4, 6, 7. [file Data_Sheet_1.pdf]
